# Genetic characterization of Yug Bogdanovac virus

**DOI:** 10.1007/s11262-012-0819-5

**Published:** 2012-10-14

**Authors:** Martin Pfeffer, Meik Dilcher, Robert B. Tesh, Frank T. Hufert, Manfred Weidmann

**Affiliations:** 1 Institute of Animal Hygiene and Veterinary Public Health, An den Tierkliniken 1, 04103 Leipzig, Germany; 2 Department of Virology, University Medical Center Goettingen, Kreuzbergring 57, 37075 Goettingen, Germany; 3 Department of Pathology, Center for Biodefense and Emerging Infectious Diseases, University of Texas Medical Branch, Galveston, TX USA

**Keywords:** Vesiculovirus, Yug Bogdanovac virus, Chandipura virus, Isfahan virus, 454 Pyrosequencing

## Abstract

We present pyrosequencing data and phylogenetic analysis for the full genome of Yug Bogdanovac virus (YBV), a member of the Vesicular stomatitis virus serogroup of the Rhabdoviridae isolated from a pool of *Phlebotomus perfiliewi* sandflies collected in Serbia in 1976. YBV shows very low nucleotide identities to other members of the Vesicular stomatitis virus serogroup and does not contain a reading frame for C′/C proteins.

Yug Bogdanovac virus (YBV) was isolated in 1976 from a pool of 200 unengorged female *Phlebotomus perfiliewi* sandflies collected in Serbia. Electron microscopy and serological analysis (complement fixation test, immunofluorescence, plaque reduction neutralization assay) placed it into the Vesicular stomatitis virus (VSV) serogroup of the Rhabdoviridae. Antibodies against YBV were found in humans (6/274 tested) [1] and domestic animals [1–3] but the role of YBV as a human pathogen is unclear. An antigenic relationship to Chandipura virus (CHDV) isolated in India and West Africa [4–7] and to Isfahan virus (ISFV) isolated in Iran and Turkmenia [8, 9], two members of the VSV serogroup implicated as causes of febrile and neurological diseases (CHDV) in humans [10–12] was described. In order to determine the genome sequence of this European vesiculovirus, YBV was passaged thrice in Vero B4 cells and RNA extraction was performed as described [13]. In order to cover the termini, a self-complimentary 3′-FLAC adapter and a 5′-RACE adapter were ligated to the genome prior to pyrosequencing [14]. A MID-barcoded Roche/454 Rapid Library was produced from 300 ng adapter-ligated viral genomic RNA following reverse transcription at 65 °C for 30 min (Transcriptor (Roche). 82 % of 11,970 reads were YBV genome specific (coverage 294-fold). The 11,202 nucleotide –ssRNA genome shows the typical genomic organization of vesiculoviruses including 3′-leader followed by 5 structural genes, and 5′-trailer sequence (Fig. 1a, GenBank JF911700). The full-length genome of YBV virus shows very low nucleotide identities to the genomes of CHDV (55.7 %), ISFV (55.3 %, [15]), VSIV (52.8 %), and of rabies virus (41.0 %) (Fig. 1b). Nevertheless, the high bootstrap values of the phylogenetic analysis confirm serological grouping of YBV within the genus *Vesiculovirus* of rhabdoviruses (Fig. 1b). In YBV, the C′/C proteins, two small highly basic, non-structural proteins encoded in a second ORF within the P gene of most vesiculoviruses [16, 17] are absent as in Alagoas virus (VSAV) [18]. Both 3′-leader and 5′-positive-sense antigenomic trailer of YBV are highly complementary to each other in the first 32 nucleotides (Fig. 1c, [19]). The genomic data presented here will help to design a YBV-specific RT-PCR which could be used to monitor YBV activity in phlebotomine sandflies in the Balkans.

**Fig. 1:**
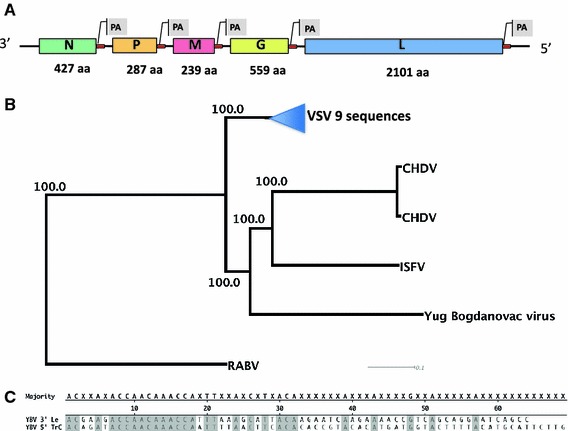
**a** YBV genome: The non-segmented negative strand 11.2 kb RNA genome sequentially encodes 5 transcription units for the nucleoprotein (N), phosphoprotein (P), matrix protein (M), glycoprotein (G), and the RNA-dependent RNA-Polymerase (L) each followed by a conserved polyadenylation signal (PA) and a short intergenic region. The amino acid sizes of the putative encoded proteins are indicated. **b** Neighbor-joining phylogenetic analysis of full genome nucleotide sequences using ClustalW and a 1,000-fold bootstrap approach rooted to the sequence of RABV (FJ712195) and collapsed VSV subtree. Bootstrap values are given in *percent*. YBV (JF911700), CHDV (GU212856, GU212858), ISFV (AJ810084), VSV (EF197793, EU849003, AF473864, AF473865, AF473866, J02428, NC_001560 (all VSIV)), EU373657 (COCV-Ind2), EU373658 (VSAV-Ind3), and RABV (FJ712195). **c** Alignment of the 3′-leader (YBV 3′ Le) and the positive-sense complement of the 5′-trailer (YBV 5′ TrC) region of YBV. Residues that match the consensus are *shaded gray*
